# Trophosome of the Deep-Sea Tubeworm *Riftia pachyptila* Inhibits Bacterial Growth

**DOI:** 10.1371/journal.pone.0146446

**Published:** 2016-01-05

**Authors:** Julia Klose, Karin Aistleitner, Matthias Horn, Liselotte Krenn, Verena Dirsch, Martin Zehl, Monika Bright

**Affiliations:** 1 Department of Limnology and Bio-Oceanography, University of Vienna, Althanstrasse 14, A-1090 Vienna, Austria; 2 Department of Microbiology and Ecosystem Science, University of Vienna, Althanstrasse 14, A-1090 Vienna, Austria; 3 Department of Pharmacognosy, University of Vienna, Althanstrasse 14, A-1090 Vienna, Austria; 4 Department of Pharmaceutical Chemistry, University of Vienna, Althanstrasse 14, A-1090, Vienna, Austria; Universite Pierre et Marie Curie, FRANCE

## Abstract

The giant tubeworm *Riftia pachyptila* lives in symbiosis with the chemoautotrophic gammaproteobacterium *Cand*. Endoriftia persephone. Symbionts are released back into the environment upon host death in high-pressure experiments, while microbial fouling is not involved in trophosome degradation. Therefore, we examined the antimicrobial effect of the tubeworm’s trophosome and skin. The growth of all four tested Gram-positive, but only of one of the tested Gram-negative bacterial strains was inhibited by freshly fixed and degrading trophosome (incubated up to ten days at either warm or cold temperature), while no effect on *Saccharomyces cerevisiae* was observed. The skin did not show antimicrobial effects. A liquid chromatography-mass spectrometric analysis of the ethanol supernatant of fixed trophosomes lead to the tentative identification of the phospholipids 1-palmitoleyl-2-lyso-phosphatidylethanolamine, 2-palmitoleyl-1-lyso-phosphatidylethanolamine and the free fatty acids palmitoleic, palmitic and oleic acid, which are known to have an antimicrobial effect. As a result of tissue autolysis, the abundance of the free fatty acids increased with longer incubation time of trophosome samples. This correlated with an increasing growth inhibition of *Bacillus subtilis* and *Listeria welshimeri*, but not of the other bacterial strains. Therefore, the free fatty acids produced upon host degradation could be the cause of inhibition of at least these two bacterial strains.

## Introduction

Since the discovery of giant tubeworms at deep-sea hydrothermal vents at the Galapagos Rift in 1977 [1], the mutualism between the sessile tubeworm *Riftia pachyptila* (Vestimentifera, Sibolinidae) (short *Riftia*) and the sulfur-oxidizing gammaproteobacterial symbiont *Cand*. Endoriftia persephone (short Endoriftia) has been one of the most extensively studied deep-sea symbioses [2]. The metagenome of Endoriftia encodes genes for sulfur oxidation and carbon fixation, but also genes for the tricarboxylic acid (TCA) cycle, fructose degradation, glycolysis as well as a phosphotransferase system and ABC transporters. The latter are indicative of a heterotrophic lifestyle that is assumed to play a role outside the host in the absence of sulfide [3]. Also genes for chemotactic abilities, including flagellar proteins, chemotaxis regulators and motility accessory factors, required for the survival outside the host were detected in the metagenome [3, 4].

Endoriftia is located in the trunk of the adult host’s body in a multi-lobule organ, the trophosome, enclosed in host cells, called bacteriocytes [5, 6, 7]. Host bacteriocytes and symbionts exhibit a coordinated cell cycle with terminal differentiation. Dividing rods in unipotent bacteriocytes acting as stem cells are located in the central zone of each lobule and small cocci and large cocci are located in semi-differentiated bacteriocytes in the median zone, while degrading large cocci in the terminal bacteriocytes of the peripheral zone enter apoptosis after digesting the symbionts [8, 9]. Nourishment of the gutless host by the symbiont is through release of fixed organic carbon [10].

Symbiont acquisition is horizontal in each host generation anew [11]. Symbionts invade the settled larvae and small juveniles as shown by fluorescence *in situ* hybridization (FISH) using three specifically designed symbiont-specific oligonucleotide probes [11]. Environmental symbionts were detected with 16S rRNA-specific PCR and FISH on artificial devices deployed in tubeworm clumps, next to clumps and far way from clumps on basalt as well as in filtered seawater from the pelagial [12].

Recently we could show in experimental high-pressure vessels that Endoriftia actively escapes dead trophosome tissue and recruits to surfaces upon which it proliferates [13]. The escape time was determined in a time series of incubations simulating either vent cessation with cold, ambient deep-sea conditions for half a day to six days or warm, hydrothermal vent conditions with a sulfide flow-through system for half a day to one day. The disintegration of the symbiont’s membranes was studied in transmission electron microscopy (TEM). These experiments revealed that under warm vent conditions most of the symbionts’ membranes were ruptured and the symbionts therefore were unambiguously dead after one day, while symbiont decay was decelerated under cold deep-sea conditions with most membranes still intact after ten days [13].

Numerous studies have shown that no other microbes colonize the trophosome in living animals apart from Endoriftia [4, 11, 14, 15, 16, 17]. Surprisingly, preliminary FISH using the symbiont-specific and the bacterial probe mix EUB338 I, II, III, which targets most bacteria simultaneously on the incubated trophosome pieces revealed no microbial fouling during host tissue degradation in our escape experiments. Therefore, we investigated whether selected Gram-positive and Gram-negative bacterial strains, or a fungus were inhibited in growth due to the presence of trophosome pieces and ethanol supernatants (derived from fixation). The trophosome samples were either freshly collected (representing the metabolism of living host and symbionts) or incubated under simulated deep-sea and hydrothermal vent conditions (representing dead host and living symbionts initially or dead host and dead symbionts at later time points). Further, we analyzed the chemical composition of the ethanol supernatants of fixed fresh and incubated trophosome samples with liquid chromatography-mass spectrometry (LC-MS) and tested whether the abundance of the identified compounds correlates with the inhibiting effect on the growth of the tested microbes.

## Materials and Methods

### Ethical statement

The research in this study is in according with the "Good Scientific Practice" of the University of Vienna. The field studies at the East Pacific Rise open ocean environment were conducted at deep-sea hydrothermal vents, which are not a private property. The cruise in 2010 to the East Pacific Rise was under the responsibility of Ifremer, with the Chief Scientist Francois Lallier. The cruise in 2011 was under the responsibility of Woods Hole Oceanographic Institute, with the Chief Scientist Scott Nooner. We further confirm, that the field studies did not involve endangered or protected species. All specimens collected were treated appropriately and just used for scientific and research purposes. The minimal amount of specimens needed for the experiments was collected.

### Sample collection and preparation

*Riftia pachyptila* tubeworms were collected in May 2010 by *R/V L’Atalante* with the submersible *Nautile* and in October 2011 by *R/V Atlantis* and ROV *Jason* at hydrothermal vents at the East Pacific Rise. Tubeworms were collected at the end of each dive, transported unpressurized to the surface within 1.5 h, dissected into trophosome and skin pieces, which were either prepared for the incubations or directly fixed in 100% ethanol or in liquid nitrogen within 15 min. To follow the degradation process of trophosome over time aboard the ship, 0.4 g (wet weight) of freshly dissected trophosome (a medium sized worm of 20 g wet weight has 3 g of trophosome, [18, 19] was incubated in high-pressure flow-through vessels at 250 bar with 0.2 μm sterile-filtered sea water at 4°C (cold condition) without flow to simulate deep-sea conditions of bottom water with 175 μmol·L^−1^ oxygen [20] and about 2–3°C at the basalt surfaces in the axial summit trough of the EPR [21]. To approximate vent habitat conditions for thriving *Riftia* [22, 23, 24] and previous maintenance conditions [25], simulated vent conditions (short warm conditions) were performed at 250 bar and a continuous flow (1 mL·min^-1^) flow at 22.4 ± 0.6°C, 280 ± 48 μmol·L^−1^ ΣH_2_S [i.e., sum of all forms of dissolved sulfide; short sulfide] [23] and 107 ± 29 μmol·L^−1^ oxygen in microporous specimen capsules for one day and up to ten days [13]. During experiments, the sulfide concentration [23], salinity and temperature were monitored continuously. As control, trophosome was fixed in 100% ethanol to kill symbionts prior to one day of high-pressure vessels incubation. Incubated trophosome was fixed in 100% ethanol or frozen in liquid nitrogen (S2 Table).

### Fluorescence in situ hybridization and transmission electron microscopy

For fluorescence *in situ* hybridization (FISH), trophosome from incubation experiments and fresh trophosome fixed in 100% ethanol was embedded in LR-White Acrylic resin medium-grade (London Resin Company Ltd.) according to [11]. For transmission electron microscopy (TEM), fresh and incubated trophosome was fixed in a mixture of 5% glutaraldehyde and 4% formaldehyde in 0.08 M sodium phosphate buffer and embedded in Low-Viscosity Resin medium-grade (Agar Scientific). 1 μm semi-thin sections for FISH and 70 nm ultrathin sections for TEM were cut using a Leica EM UC7 ultramicrotome. FISH on semi-thin section was performed according to Nussbaumer *et al*. (2006). Sections were hybridized simultaneously with the symbiont-specific probe RifTO445 [11] labeled in either FITC or Cy3 and the general bacterial probe mix EUB338 I, II, III labeled in either Cy3 or FITC (S1 Table). The nonsense probe NON-388 was used with the same fluorescence label as the probes on each slide of the treatments separately as negative control. 4',6-Diamidino-2-phenylindole (DAPI) was used as counterstain. Microscopic analyses were performed with a Zeiss Axio Imager epifluorescence microscope. For TEM investigations, ultrathin sections were stained with uranyl acetate for 25 min or gadolinium for 15 min and lead citrate for 7 min and analyzed with a Zeiss EM 902 transmission electron microscope.

### Antimicrobial bioassay

Growth inhibition by freshly fixed and incubated trophosome and freshly fixed skin material was tested for the following organisms: *Bacillus subtilis* (Firmicutes), grown in lysogeny broth (LB) at 37°C; *Listeria welshimeri* (Firmicutes), grown in brain-heart infusion (BHI) at 37°C; *Mycobacterium smegmatis* (Actinomycetales), LB at 37°C; *Staphylococcus aureus* (Firmicutes), LB at 37°C as representatives of Gram-positive bacteria; *Vibrio cholerae* (Gammaproteobacteria), LB 37°C; *Burkolderia cepacia* (Betaproteobacteria), LB 37°C; *Flavobacterium johnsoniae* (Bacteroidetes), LB 30°C; *Escherichia coli* (Gammaproteobacteria), LB 37°C as representatives of Gram-negative bacteria, and the eukaryote *Saccharomyces cerevisiae* (Saccharomycetaceae), Yeast Peptone Dextrose (YPD) 30°C. Prior to plating, bacterial cultures were grown over night to the logarithmic phase and spread equally on plates. To test for growth inhibition of bacterial strains, 0.08 g frozen skin, 0.08 g freshly fixed or incubated frozen trophosome or trophosome homogenized in 50 μL 0.1M sodium phosphate buffer (PBS) pH 7.4 was placed on freshly plated BHI or LB agar medium plates and incubated at the indicated temperature over night. Each of the experiments was carried out at least twice. Additionally, the ethanol supernatant of fixed trophosome was evaporated in a Concentrator plus Vacufuge^®^ (Eppendorf). The residue was mixed with 20 μL PBS. Filter discs soaked with 10 μL of this solution were used for inhibition assays as described above. As controls, 1 μL of ampicillin dissolved to different concentrations in PBS (100, 20, 10, 5, and 2 mg/mL) and PBS without antibiotic (vehicle control) were used on each of the plates. Results were documented photographically and the zone of inhibition was measured on the photographs. We used three individual plates for each trophosome piece, incubation experiment and bacterial strain tested. Different tubeworm specimens were used in each incubation experiment. One plate per bacterial strain was used to test for the inhibitory effect of freshly fixed skin (S2 Table).

### High-performance liquid chromatography—mass spectrometry

To identify the potential antimicrobial compounds in the trophosome, the ethanol supernatant of the fixed, fresh and incubated trophosome samples and the ethanol supernatant of the skin were analyzed by high-performance liquid chromatography (HPLC) with charged aerosol detection (CAD) and HPLC-mass spectrometry (MS). The CAD is a universal detector that serves to examine relative quantities of the non- and semi-volatile constituents [26]. These analyses were performed on an UltiMate 3000 RSLC-series system (Dionex/Thermo Fisher Scientific, Germering, Germany) coupled in parallel to a Corona ultra RS charged aerosol detector (CAD, Dionex/Thermo Fisher Scientific) and an HCT 3D quadrupole ion trap mass spectrometer equipped with an orthogonal ESI source (Bruker Daltonics, Bremen, Germany). Separation was carried out on an Acclaim 120 C18, 2.1 x 150 mm, 3 μm HPLC column (Dionex/Thermo Fisher Scientific) using 0.1% aqueous formic acid and acetonitrile as mobile phase A and B, respectively. Gradient elution started with a 2 min isocratic step with 5% B, followed by a linear increase to 95% B in 45 min, and finally a column cleaning and re-equilibration step. The flow rate was 0.5 mL/min and the column oven temperature was set to 25°C. After passing the DAD, the eluate flow was split 4:1 between the CAD and the MS, respectively. The CAD nebulizer temperature was 35°C and the ESI ion source was operated as follows: capillary voltage: +3.5/-3.7 kV, nebulizer: 26 psi (N_2_), dry gas flow: 9 L/min (N_2_), and dry temperature: 340°C. Low-energy collision-induced dissociation (CID) mass spectra were obtained in time-scheduled experiments using helium as collision gas, an isolation window of Δ*m/z* = 2, and a fragmentation amplitude of 0.7 V.

To confirm the tentative identifications achieved with the above system, high-resolution mass spectra were recorded on a maXis HD ESI-Qq-TOF mass spectrometer (Bruker Daltonics) that was also connected to an UltiMate 3000 RSLC-series system. The separation of one typical sample was performed with the above described HPLC method and the following ESI ion source settings were applied: capillary voltage: ±4.5 kV, nebulizer: 2.0 bar (N_2_), dry gas flow: 8.0 L/min (N_2_), and dry temperature: 200°C. The sum formulas of the detected ions were determined using Bruker Compass DataAnalysis 4.2 based on the mass accuracy (Δ*m/z* ≤ 2 ppm) and isotopic pattern matching (SmartFormula algorithm). The freshly fixed trophosome was analyzed in triplicates from three different specimens. For the incubated trophosome, the ethanol supernatant from one experiment and one specimen was analyzed. For the mass spectrometric analysis we used the ethanol supernatants of three specimens (S2 Table).

### Statistical analysis

The peak areas of each of the five lipid compounds as obtained by HPLC-CAD analyses of the ethanol supernatants of freshly fixed and incubated trophosome and of the skin were correlated to the measured inhibition zone in the assay (S1 Data). To determine correlations between the lipid compounds and the inhibitory effect of the freshly fixed and incubated trophosome and freshly fixed skin, Spearman correlation coefficients with significance levels (*** p < 0.001, ** p < 0.01 and * p < 0.05) were calculated in R.

## Results

**Fig 1:**
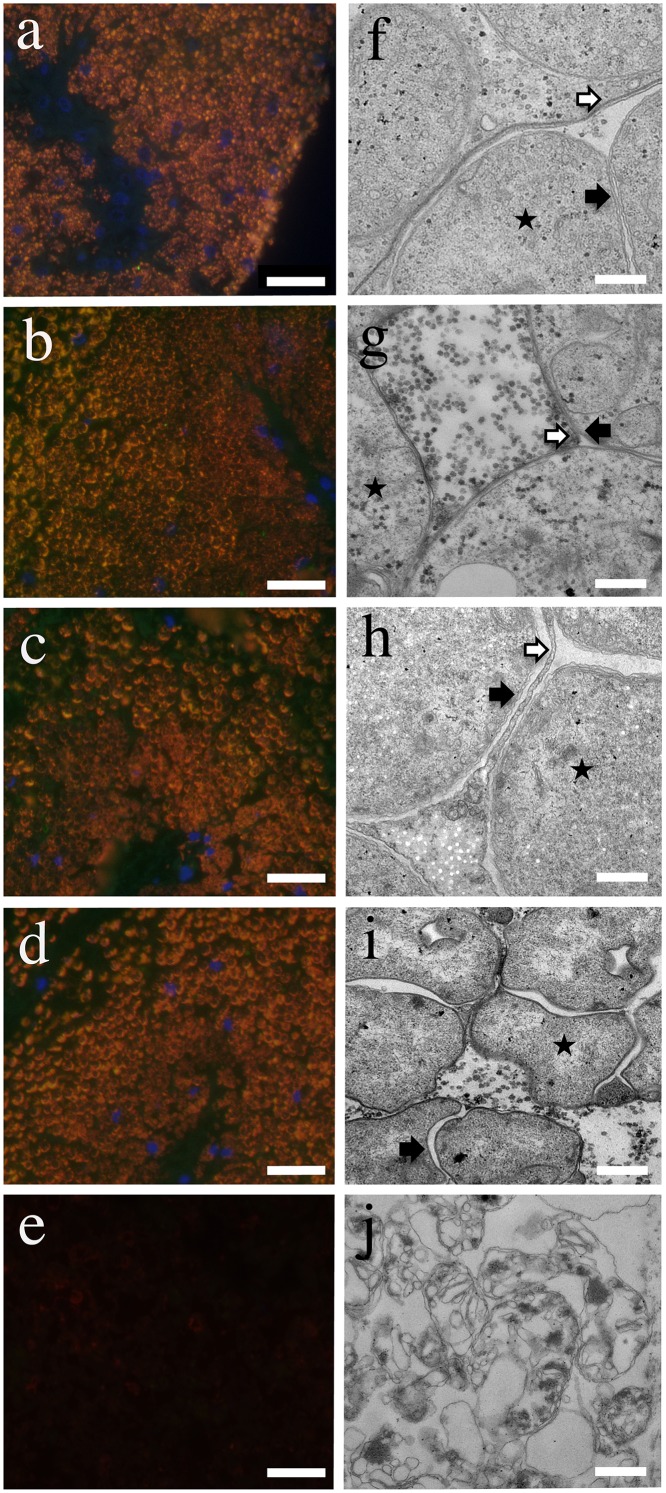
Decay of fresh and incubated trophosome over time under cold and warm incubation conditions. a, f) Fresh trophosome, b, g) one day cold incubated trophosome, c, h) six day cold incubated trophosome, d, i) one day warm incubated trophosome, e, j) six day warm incubated trophosome. a-e) Fluorescence *in situ* hybridization (FISH) on LR-White sections of fresh and incubated trophosome shows a perfect overlap of signals for the symbiont specific and general bacterial probe mix. Red: EUB I, II, III mix; green: symbiont probe RifTO445; blue: DAPI; scale bars: 20 μm. f-j) Transmission electron micrographs (TEM) of fresh and incubated trophosome. A star indicates symbiont presence in the trophosome; black arrow: symbiont outer and cell membrane; white arrow: host symbiosome membrane; scale bars: 500 nm.

All bacterial cells in the trophosome of freshly collected animals were simultaneously labeled with the general EUB probe mix, targeting most bacteria, the symbiont-specific probe, and DAPI. The results confirmed earlier studies that found no other microbes than Endoriftia living in the trophosome [11, 14, 15, 17]. Surprisingly, however, when the trophosome was incubated for up to six days under cold conditions (simulating the deep-sea) and warm conditions (simulating hydrothermal vents), still no other microbes were found to colonize the decaying host tissue (Fig 1a–1e), despite the fact that about 10^3^−10^5^ prokaryotes were present in 1 mL of incubation water as determined by general bacterial probe mix and DAPI counts.

The symbiont-specific and the general EUB probe mix signals were still positive in the trophosome sections obtained after cold incubation for six days (Fig 1c), while there were no FISH and DAPI signals after six days of warm incubation (Fig 1e, S1 Table). Trophosomes fixed in ethanol to kill both, host and symbiont, prior to incubations did not show a FISH or DAPI signal after one day of cold incubation. These findings go hand in hand with symbiont degradation during experimental incubations [13]. Freshly fixed specimens exhibited intact symbiont outer, cytoplasmic, and sulfur vesicle membranes in TEM sections (Fig 1f). The membrane integrity of symbionts decreased with the time of incubation, albeit at different time scales under cold and warm conditions. After one day or six days of cold incubation as well as one day of warm incubation, symbiont membranes were still intact (Fig 1g–1i). In contrast, after six days of warm incubation, the entire symbiont tissue including the membranes was mostly disintegrated (Fig 1j). Differences in the rate of degradation between the dead host tissue and the symbiont were clearly discernible by TEM, which revealed disintegrated symbiosome membranes of host origin, but mostly intact symbiont membranes after six days of cold incubations and after one day of warm incubations. This indicates that the symbionts died during the warm incubations between one and six days, while during cold incubations they remained alive up to six days.

**Fig 2:**
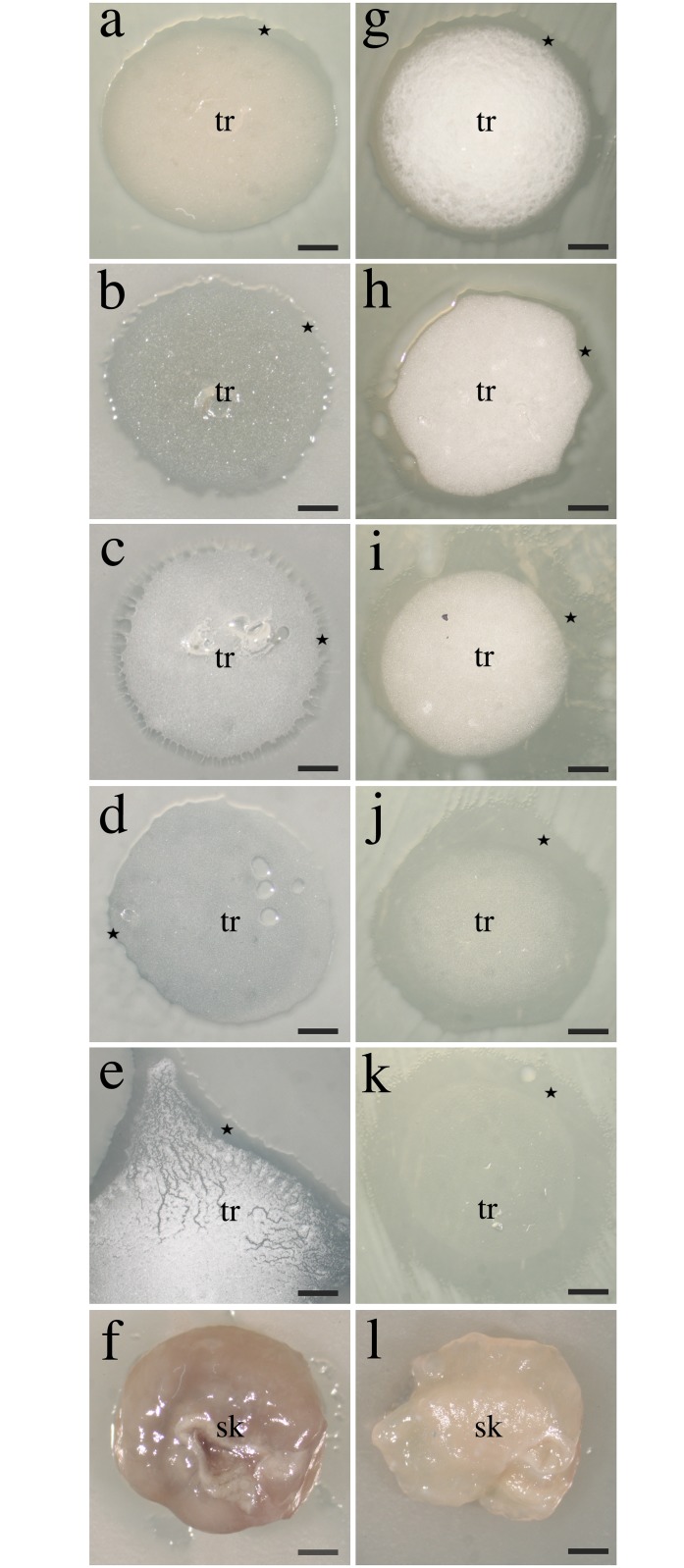
Agar diffusion test for the inhibition of bacterial growth by freshly fixed and gradually degraded trophosome and freshly fixed skin. Inhibition of growth of a-f) *Bacillus subtilis* and g-l) *Listeria welshimeri* by a, g) freshly fixed trophosome, b, h) one day cold incubated trophosome, c, i) six days cold incubated trophosome, d, j) one day warm incubated trophosome and e, k) six days warm incubated trophosome. f, l) No growth inhibition of *Bacillus subtilis* and *Listeria welshimeri* by freshly fixed *Riftia* skin. The inhibition of both strains by the trophosome correlates significantly with the peak areas of the free fatty acids from the HPLC-CAD analysis. Star indicated zone of inhibition, tr: trophosome, sk: skin, scale bars: 1 mm.

**Fig 3:**
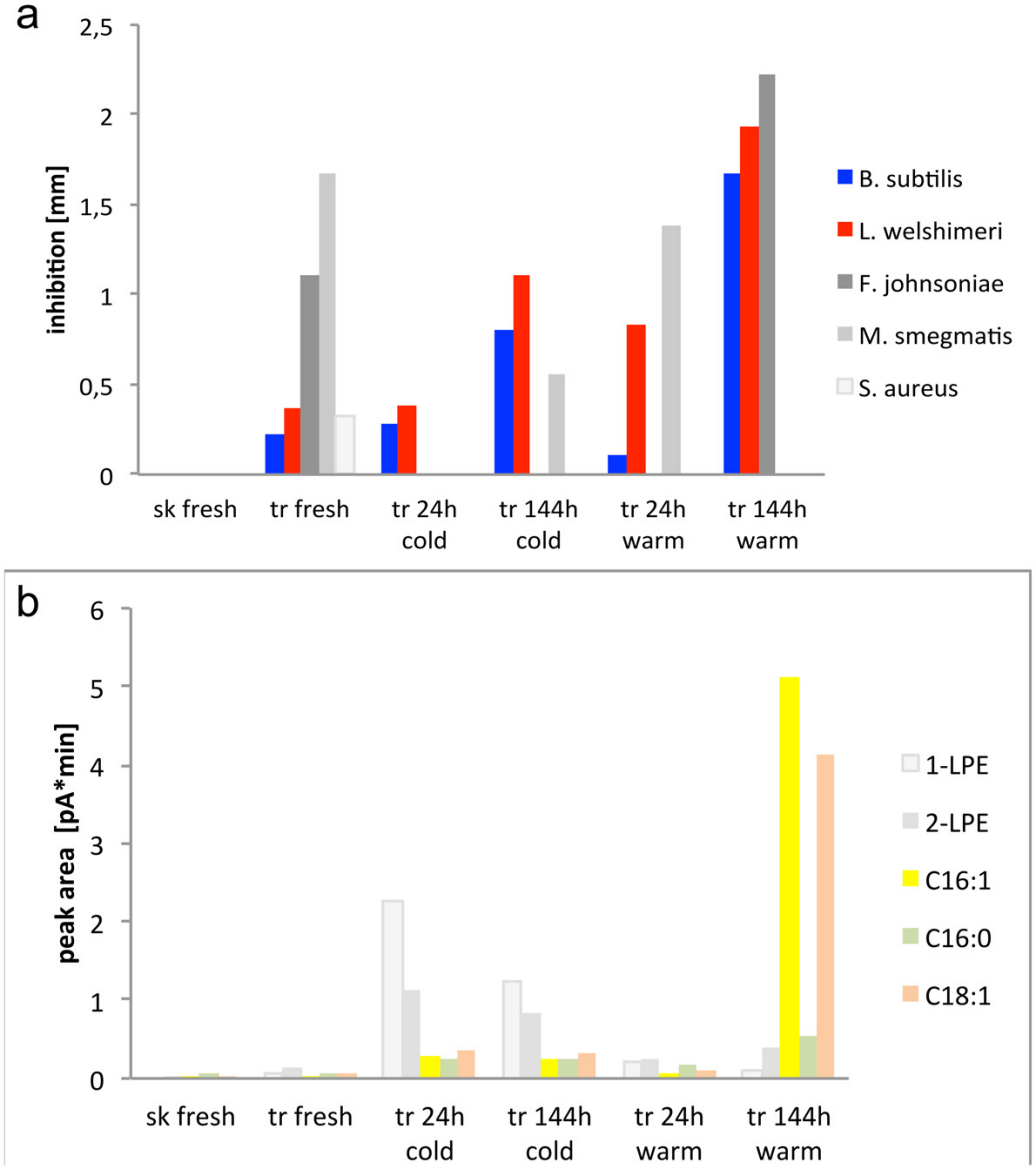
**a)** Measured inhibition zones in mm induced by freshly fixed and incubated (cold and warm) trophosome samples and freshly fixed skin on *Bacillus subtilis*, *Listeria welshimeri*, *Flavobacterium johnsoniae*, *Mycobacterium smegmatis* and *Staphylococcus aureus*. **b)** Absolute peak areas of the lipids 2-palmitoleyl-1-lyso-PE (1-LPE), 1-palmitoleyl-2-lyso-PE (2-LPE), palmitoleic acid (C_16_:1), palmitic acid (C_16_:0), and oleic acid (C_18_:1) obtained by HPLC-CAD analysis of the corresponding ethanol supernatants. The correlation of the inhibition zone of *Bacillus subtilis* and *Listeria welshimeri* with the peak areas of the three free fatty acids is highlighted with the colours. sk: skin, tr: trophosome (S3 Table).

To assess the antimicrobial effect of the trophosome, we performed an antimicrobial assay. Several bacteria (*Vibrio cholerae*, *Escherichia coli*, *Burkolderia cepacia)* and the fungus *Saccharomyces cerevisiae* were not inhibited in growth in the presence of freshly fixed trophosome and skin pieces. Freshly fixed trophosome, however, inhibited the growth of the Gram-positive bacteria *Bacillus subtilis*, *Listeria welshimeri*, *Mycobacterium smegmatis* and *Staphylococcus aureus* and the Gram-negative *Flavobacterium johnsoniae*, assessed by the presence of inhibition zones of 0.11–3.06 mm in antimicrobial assays (S3 Table). Fig 2 displays the inhibition of *Bacillus subtilis* and *Listeria welshimeri* by the freshly fixed trophosome (Fig 2a and 2g, respectively), by the trophosome after one day of cold incubation (Fig 2b and 2h), after six days of cold incubation (Fig 2c and 2i), after one day of warm incubation (Fig 2d and 2j) and after six days of warm incubation (Fig 2e and 2k). Also ethanol supernatant samples (i.e., the ethanol used to preserve the trophosomes), after evaporation of the solvent and dissolution in phosphate buffered saline (PBS), showed the same inhibitory effect on bacterial growth as the trophosome for all samples analyzed, indicating the presence of ethanol-soluble antimicrobial compounds in the trophosome. The inhibition of bacterial strains varied in relation to incubation time and conditions (Fig 3, S3 Table). Freshly fixed skin pieces were not inhibiting the growth of these strains, which is displayed in Fig 2f and 2l for *Bacillus subtilis* and *Listeria welshimeri*, respectively.

For all antimicrobial bioassays, three different controls were conducted: 1) Ethanol-fixed samples of trophosome prior incubation (with dead host and dead symbiont) inhibited the growth of *Bacillus subtilis* after half a day and one day of incubation under cold, high pressure conditions, while none of the other strains was inhibited in growth. Additionally, pure ethanol was tested for inhibition of the growth but did not inhibit any of the strains tested. 2) We tested whether PBS alone inhibits growth and found no inhibition on any strain, while 3) the PBS control with ampicillin showed an inhibition of bacterial growth on all strains tested.

**Fig 4:**
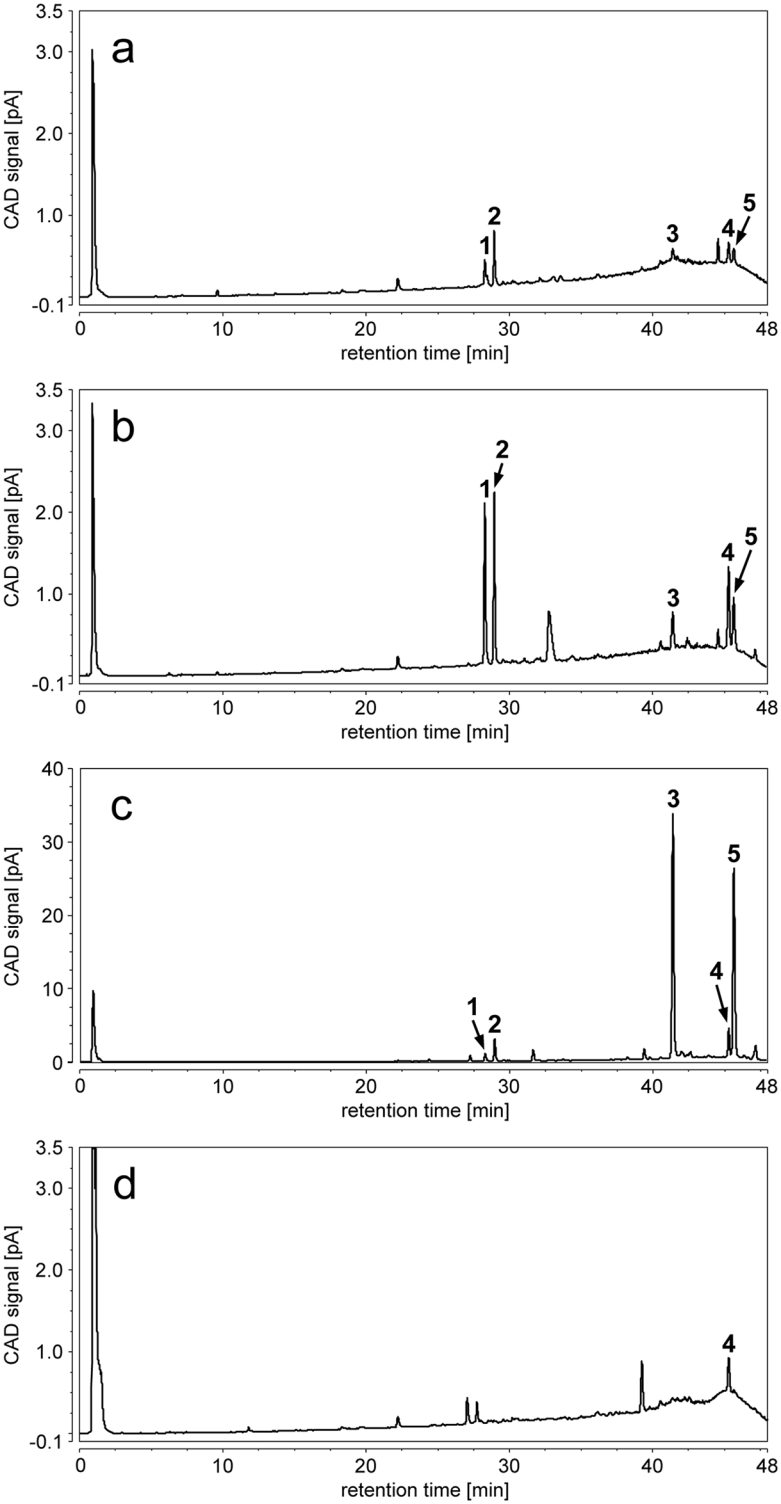
HPLC-CAD chromatograms of the ethanol supernatant after fixation of a) freshly fixed trophosome, b) trophosome after one day warm incubation, c) trophosome after six days warm incubation, and d) freshly fixed skin of *Riftia pachyptila*. Peaks **1**–**5** were tentatively identified by HPLC-MS as 2-palmitoleyl-1-lyso-PE (1-LPE), 1-palmitoleyl-2-lyso-PE (2-LPE), palmitoleic acid, palmitic acid, and oleic acid, respectively.

Since the ethanol supernatants showed comparable antimicrobial effects to the respective tissue samples, we analyzed the former by LC-MS to identify the constituents responsible for this activity. All ethanol supernatants of freshly fixed and incubated trophosome samples showed an abundant pair of compounds eluting at 28.3 min and 29.0 min (Fig 4a–4d). Based on the typical fragmentation pattern and the sum formula of C_21_H_42_NO_7_P obtained by high-resolution mass spectrometry (S1 Data), these two main constituents were identified as lysophosphatidylethanolamines (C_16:1_), with the first peak most likely being 1-hydroxy-2-palmitoleyl-sn-glycero-3-phosphoethanolamine (2-palmitoleyl-1-lyso-PE, 1-LPE) and the second peak most probably corresponding to 1-palmitoleyl-2-hydroxy-sn-glycero-3-phosphoethanolamine (1-palmitoleyl-2-lyso-PE, 2-LPE) [27, 28, 29, 30]. In addition, free fatty acids—mainly palmitoleic acid (C_16:1_), palmitic acid (C_16:0_), and oleic acid (C_18:1_)—were detected in freshly fixed and incubated trophosome samples (Fig 4a–4c). In contrast, LPEs, palmitoleic acid and oleic acid were detected only in very low abundance in ethanol supernatants of three freshly fixed skin samples (Fig 4d).

A comparison of the peak areas of the LPEs and fatty acids, obtained by HPLC-CAD analysis of the ethanol supernatants (Fig 3, S3 Table), between treatments of trophosome tissue revealed positive correlations between contents of 1-LPE and 2-LPE, between 2-LPE, palmitoleic acid and oleic acid, and between all three fatty acids (Table 1). Overall, while all five identified components were already present in the freshly fixed trophosome, their abundance increased upon incubation. The content of LPEs was higher after cold compared to warm incubation, particularly after one day cold incubation. In contrast, the free fatty acids became highly abundant upon incubation for six days under warm conditions, whereby the content of monounsaturated fatty acids showed a stronger increase (Figs 3 and 4c, S3 Table).

**Table 1:** Spearman correlation coefficients between the peak areas of 2-palmitoleyl-1-lyso-PE (1-LPE), 1-palmitoleyl-2-lyso-PE (2-LPE), palmitoleic acid (C_16_:1), palmitic acid (C_16_:0), and oleic acid (C_18_:1) and the growth inhibitory effect of freshly fixed and incubated trophosome and skin on *Bacillus subtilis*, *Listeria welshimeri*, *Flavobacterium johnsoniae*, and *Mycobacterium smegmatis*. Correlations are between 1-LPE and 2-LPE, between 2-LPE, palmitoleic acid and oleic acid, and between all three fatty acids. The peak areas of all three fatty acids correlate with the inhibition of *Bacillus subtilis* and *Listeria welshimeri*.

	*B*. *subtilis*	*L*. *welshimeri*	*F*. *johnsoniae*	*M*. *smegmatis*	1-LPE	2-LPE	C16:1	C16:0
*L*. *welshimeri*	0.83*							
*F*. *johnsoniae*	0.51	0.34						
*M*. *smegmatis*	-0.21	-0.09	0.07					
1-LPE	0.49	0.49	-0.37	-0.09				
2-LPE	0.71	0.60	-0.14	-0.27	0.94**			
C16:1	0.89*	0.83*	0.34	-0.39	0.66	0.83*		
C16:0	0.94**	0.94**	0.34	-0.27	0.60	0.77	0.94**	
C18:1	0.89*	0.83*	0.34	-0.39	0.66	0.83*	1.00***	0.94**

Significance levels are:

*** p < 0.001,

** p < 0.01 and

* p < 0.05.

The peak areas of all three detected fatty acids, namely palmitoleic acid (C_16:1_), palmitic acid (C_16:0_), and oleic acid (C_18:1_) correlate with the inhibition of *Bacillus subtilis* and *Listeria welshimeri* (p < 0.05 for C_16:1_ and C_18:1_, p < 0.01 for C_16:0_) (Table 1). The growth inhibition induced by freshly fixed trophosomes corresponds to a zone of inhibition of 0.22 mm diameter for *Bacillus subtilis* and to 0.37 mm diameter for *Listeria welshimeri*. The zone of inhibition for *Bacillus subtilis* increased to 0.28 mm and 0.80 mm diameter when testing the trophosome after one day and six days of cold incubation, respectively. In the case of warm incubation, the zone of inhibition was 0.11 mm after one day, but showed a pronounced increase to 1.67 mm for six days incubated trophosome. The zone of inhibition for *Listeria welshimeri* was 0.39 mm and 1.11 mm for one day and six days cold incubated trophosome, respectively, and was more pronounced with 0.83 mm and 1.94 mm in one day warm and six days warm incubated trophosome, respectively. In correlation, a low peak area for the free fatty acids corresponding to a low content in the freshly fixed trophosome, with an increase in peak area for the cold incubated trophosome and a strong increase for the six days warm incubated trophosome, was found. The skin, which showed very low peak areas for all three fatty acids, was not inhibiting growth of any of the strains tested (Fig 3, S3 Table).

No correlation between the content of LPEs and the inhibitory effect on the strains *Bacillus subtilis* and *Listeria welshimeri* was detected. Likewise, no correlation with any lipid compounds and the inhibited bacterial strains *Flavobacterium johnsoniae* and *Mycobacterium smegmatis* was found (Table 1, Fig 3, S3 Table).

## Discussion

*Riftia* is one of the fastest growing invertebrates we know of [31]. This requires a metabolically highly active host supplying the symbiont with molecular carbon dioxide, sulfide, oxygen, and nitrogen—the latter mainly in the form of nitrate and ammonium—for the symbionts to be chemoautotrophically active [2, 3, 32]. Fixed carbon not only serves the symbionts’ growth but also nourishes the gutless host [8, 10, 33].

While this nutritional interplay leads to proliferation rates as high as observed in cancer cells or wound healing processes [9], at the same time the host controls the population density of the symbiont in a cell cycle with terminal differentiation. Growth of the trophosome tissue occurs through stem cells in the center of each lobule and leads to new lobules as well as to the renewal of bacteriocytes that cycle from the center towards the periphery of each lobule where apoptosis occurs. Therefore, the trophosome tissue exhibits not only high proliferation rates but also relatively high apoptosis rates. In addition, symbionts are continuously digested in the periphery and replaced by dividing symbionts in the center [9].

The detection of relatively high amounts of lysophosphatidylethanolamines and fatty acids in the trophosome may reflect the high turnover of host and symbiont cells in the trophosome, with cell death resulting in degradation of tissue and membranes. While LPEs are present in small quantities in eukaryote and bacterial membranes [27, 34], low free fatty acid concentrations are indicative for low phospholipid breakdown and low enzymatic and lipolytic activity [35, 36]. During natural degradation of membranes, LPEs and free fatty acids are products of phospholipid hydrolysis by phospholipases [28].

Differences in amounts of LPEs and fatty acids in the freshly fixed trophosome and freshly fixed skin of *Riftia* may be explained by the different cell kinetics. Renewal of the skin tissue is accomplished by high proliferation and little apoptosis leading to fast growth [9]. The lack of symbionts in the skin as well as little host cell death consequently results in low amounts of LPEs and fatty acids detected in the skin.

The lipid composition of *Riftia* detected in this study is very similar to the lipid composition of the close relative *Ridgeia piscesae* with the fatty acids C_16:0_, C_16:1_ and C_18:1_ (found in trophosome and plume in *Ridgeia*) and it is characteristic for a bacteria-based diet of these hydrothermal vent invertebrates [37, 38]. The two sulfur-oxidizing bacterial markers C_16:1_ and C_18:1_ occur in high amount in the skin and plume of *Riftia* and are indicative that the nutrition of *Riftia* is based on the translocation of fixed carbon from thiotrophic bacteria to the host [35, 39] Relatively high levels of phospholipids and also sterols point to a membrane structure function in both tubeworm species rather than to a role as energy reserve [35], being consistent with a relative constant food source through thiotrophic bacteria [40]. Furthermore, no other LPEs (e.g., C_16:0_, C_18:0_, C_18:1_) than C_16:1_-LPEs were detected in the trophosome of *Riftia*, which might point to a special lipid composition in the trophosome or the presence of specific enzymes, which exclusively produce the LPEs found.

Importantly, the five lipid compounds analyzed in this study do not represent the full range of trophosome’s lipid composition, since only the least lipophilic ones are detectable with the employed HPLC method. A more comprehensive analysis of the lipid composition, including sterols and triacylglycerols, was shown for *Riftia* in [35] and for the gutless siboglinid *Oligobrachia mashikoi* in [41]. Nevertheless, all fatty acids found in the *Riftia* trophosome in this study, as well as phosphatidylethanolamines, are also present in the *Oligobrachia mashikoi* trophosome [41]. This beardworm also harbors sulfur-oxidizing symbionts but in a very simple, two-layered trophosome with few symbionts only [42, 43, 44]. The lipid composition of the trophosome was similar to the lipid composition in the skin of *Oligobrachia mashikoi* [41], which might be explained by the lower symbiont content in the trophosome compared to the one in *Riftia*.

We performed our experiments to investigate the escape of the symbiont from dead host trophosome tissue. We found that the symbiont was alive for about one day but died between day one and day six under warm vent conditions (Fig 1). In contrast, little signs of symbiont death were detected within six days of incubations under cold conditions. Decomposition of eukaryotic tissue through autolysis, the self-digestion by endogenous enzymes, begins within minutes after the death without bacterial influence [45, 46]. Accumulation of waste products with cell and lysosomal membrane disintegration, results in the release of enzymes (proteotylic, lipolytic, glycolytic) into the cytoplasm and subsequently in the breakdown of e.g., lipids [45, 47, 48]. The cell membrane releases nutrients, including fatty acids, as an energy and food source for bacteria and thus facilitates putrefaction [45, 48]. Bacterial decomposition of tissue with an increase in autolysis and putrefaction [48] was shown for tissue stored at 30°C, while refrigeration decelerated these processes [49].

**Fig 5:**
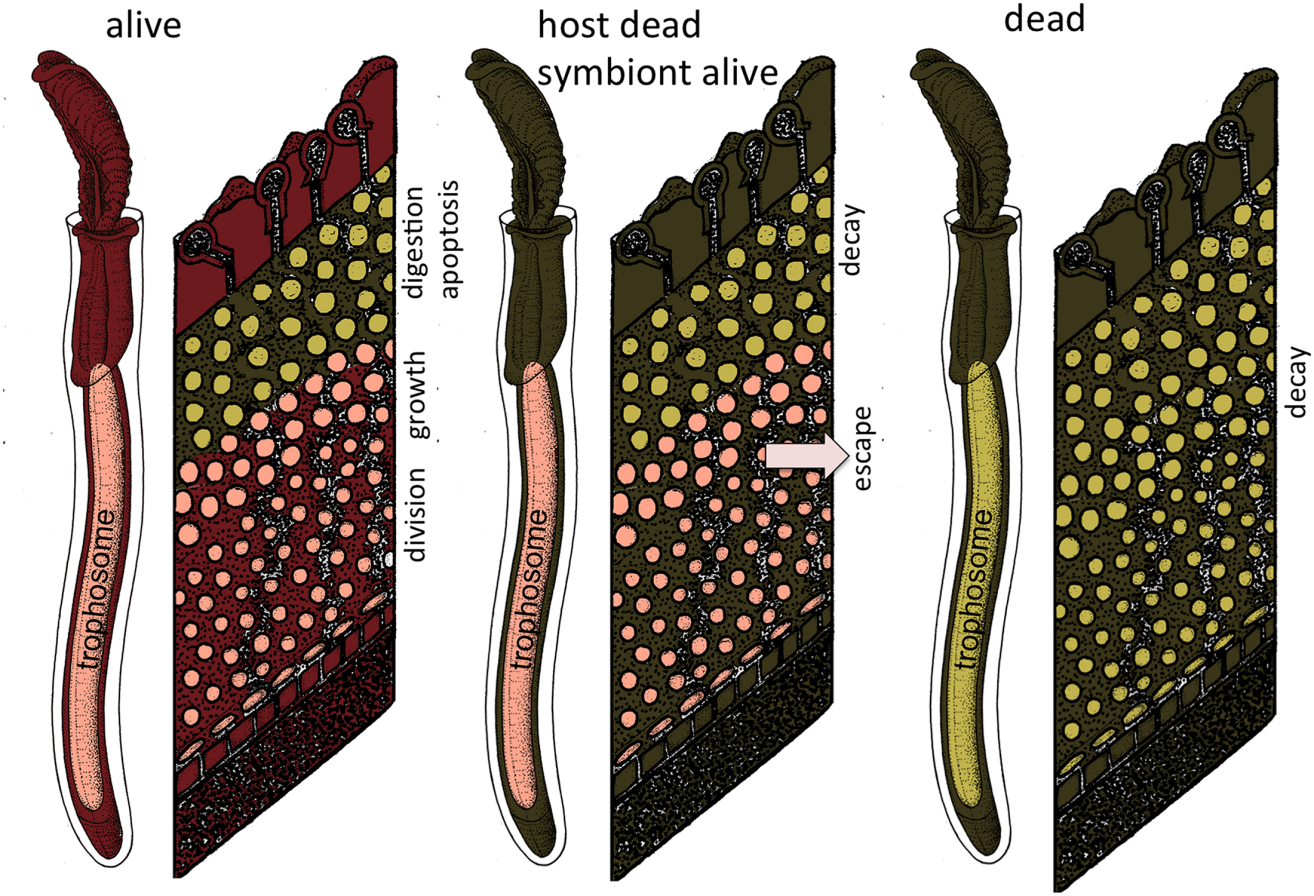
Symbiont viability in the course of host degradation. In fresh specimens, the host and symbiont are alive and the trophosome and skin samples represent the lipid composition of the holobiont. Upon host death, i.e., after trophosome incubation for one day of cold and warm incubations and after six days of cold incubations in our experiments, symbiont digestion ceases. The *in situ* membrane degradation of symbionts quantitatively exceeds the degradation of host membranes upon host death, and therefore LPEs and fatty acids decrease. Symbiont and remaining host autolysis between day one and day six of warm incubations results in an increase of the concentration of free fatty acids. Red: host alive; pink: symbiont alive; dark green: host dead; light green: symbiont dead.

Our ultrastructural analysis allows us to formulate a hypothesis on symbiont viability in the course of host degradation, i.e., in relation to LPEs and fatty acid concentration and in comparison to autolytic processes studied in forensic science (Fig 5): 1) Initially, host death results in degradation of host membranes and should theoretically increase the concentration of LPEs and free fatty acids. However, due to host death, digestion of symbionts also ceases. We hypothesize that the *in situ* membrane degradation of symbionts under production of LPEs and fatty acids quantitatively exceeds the degradation of host membranes upon host death, and therefore these compounds decrease after one day of cold and warm incubations as well as after six days of cold incubations. 2) Upon symbiont death between day one and day six of warm incubations, not only the remaining host undergoes autolysis, but also the symbionts. Therefore LPEs and fatty acid concentrations increase.

It has been long known that no other microbes except Endoriftia live in the trophosome of adult tubeworms [4, 14, 15, 16, 17]. To our surprise, however, no microbes were found to colonize dead trophosome tissue under warm as well as cold conditions for up to six days despite the fact that plenty of marine prokaryotes were present in our incubation water.

Our inhibition experiments support previous findings of antimicrobial effects of free fatty acids, however, also revealed that: 1) Some microbes, like *Vibrio cholerae*, *Escherichia coli*, *Burkolderia cepacia* and the eukaryote *Saccharomyces cerevisiae* were not inhibited by the trophosome tissue nor by the dried residue of ethanol supernatants after their fixation, 2) The bacteria *Flavobacterium johnsoniae*, *Mycobacterium smegmatis* and *Staphylococcus aureus* were inhibited in growth. However, no correlation between growth inhibition and abundance of LPEs or fatty acids could be detected. Thus, there is currently no indication how this inhibition was accomplished and further analyses need to be carried out. 3) Inhibition efficacy against *Bacillus subtilis* and *Listeria welshimeri* correlated with the abundance of the detected fatty acids. This indicates that the antimicrobial activity against these two bacterial strains might be indeed due to the amount and nature of fatty acids produced in the course of initially host degradation alone and later host and symbiont degradation.

Overall, these antimicrobial effects upon host death may support Endoriftia to escape the dead host. Due to their amphiphilic properties, lysophospholipids have an antifouling effect, which can prevent biofilm formation [50] and potentially bacterial overgrowth on sessile tubeworms. In general, LPEs and fatty acids are known to have antimicrobial effects. While an antimicrobial and antifungal effect of 1-LPE (C_16:1_) is described in the housefly larvae *Musca domestica* [28], the antimicrobial and antifungal effect of fatty acids was shown in several studies [51, 52, 53, 54] The lysophospholipid-dependent mechanism for selective inhibition mainly of Gram-positive and not of Gram-negative bacteria is supposed to be mediated through an inhibitory effect on bacterial K^+^-transport systems [55]. Free fatty acids target the cell membrane and are responsible for the disruption of the electron transport chain [56, 57, 58]. Further they also influence the oxidative phosphorylation and therefore interfere with the cellular energy production [59, 60]. Gram-negative bacteria are generally more resistant to medium- and long-chain fatty acids and their derivatives than Gram-positive strains due to their cell wall lipopolysaccharides preventing lipids to accumulate in cell membrane and subsequently to enter the cell [51, 52, 61, 62, 63]. The growth of the Gram-positive bacteria *Staphylococcus aureus*, *Carnobacterium piscicola*, *Lactobacillus curvatus*, and *Lactobacillus sake* was inhibited by palmitoleic acid, while palmitic acid and oleic acid did not have any effect on these and several other bacterial strains, including the Gram-negative bacteria *Brochothrix thermosphacta*, *Pseudomonas fluorescens* and *Serratia liquefaciens* [51, 53]. These results go hand in hand with the results of this study, where the majority of Gram-positive bacteria was inhibited, while the only Gram-negative bacterium that was inhibited in growth by trophosome was *Flavobacterium johnsoniae*.

As no or only few other microbes colonize dead host tissue, Endoriftia faces less or no competition during this critical process. Therefore, the lipid compounds detected in our study, which are likely derived from natural tissue autolysis, might support the release of symbionts into the ambient environment upon host death. Further, the symbiont is no longer actively supplied with any nutrients for carbon fixation from the host after its death. The symbiont might still potentially receive sulfide from tissue degradation and is able to fix carbon as long as oxygen is not depleted. Further, it might be speculated that under these conditions Endoriftia switches to a heterotrophic lifestyle and feeds on the dead host until it escapes. Post mortem processes in eukaryotes include also putrefaction with bacterial hydrolysis of triglycerides resulting in a mixture of free fatty acids [64, 65]. Whether Endoriftia plays an active role in the decomposition of the trophosome remains to be studied.

## Supporting Information

S1 Data Mass spectrometric data of the tentatively identified compounds. (DOCX)

S1 Table Oligonucleotide probes for fluorescence in situ hybridization (FISH). Probes were labeled on the 5’ end either with Cy3, FITC or Atto 488 to highlight symbionts in the trophosome and to analyze other bacteria on the tissue-sections. A NON-EUB338 probe was used as negative control. All hybridizations were carried out in 35% (vol/vol) formamide and counter-stained with DAPI. The Endoriftia oligonucleotide probe RifTO445 (Nussbaumer et al., 2006) is specific for the 16S rRNA of the *Riftia pachyptila*, *Tevnia jerichonana* and *Oasisia alvinae* symbionts as all three vestimentiferans share an identical (*Tevnia*) or nearly identical (*Oasisia*) 16S rRNA symbiont phylotype (*Rif/Tev/Oas*) (Feldman et al., 1997; Vrijenhoek et al., 2010; Gardebrecht et al., 2012).
(DOCX)

S2 Table Samples of freshly fixed, cold incubated and warm incubated trophosome and of freshly fixed skin from *Riftia* for chemical analyses using HPLC-CAD and HPLC-MS analyses and for inhibition assays. TM: trophosome middle part, SM: skin middle part, SU: skin upper part, cryo: fixed in liquid nitrogen, ethanol: fixed in 100% ethanol.
(DOCX)

S3 Table Measured inhibition zones in mm induced by freshly fixed and incubated (cold and warm) trophosome samples and freshly fixed skin on *Bacillus subtilis*, *Listeria welshimeri*, *Flavobacterium johnsoniae*, *Mycobacterium smegmatis* and *Staphylococcus aureus*. Absolute peak areas of the lipids 2-palmitoleyl-1-lyso-PE (1-LPE), 1-palmitoleyl-2-lyso-PE (2-LPE), palmitoleic acid (C_16_:1), palmitic acid (C_16_:0), and oleic acid (C_18_:1) obtained by HPLC-CAD analysis of the corresponding ethanol supernatants. N/A: data not available.
(DOCX)
