# Supplementary material for: Trophosome of the Deep-Sea Tubeworm Riftia pachyptila Inhibits Bacterial Growth
Source: PLoS One. 2016 Jan 5;11(1):e0146446. doi: 10.1371/journal.pone.0146446 (PMC4701499; doi:10.1371/journal.pone.0146446)
Supplement: S1 Data — (DOCX) [file pone.0146446.s001.docx]

**1-Hydroxy-2-palmitoleyl-sn-glycero-3-phosphoethanolamine (1-LPE) (1):** +ESIMS (Electrospray ionization mass spectrometry) *m/z* 452.2 [M+H]^+^; +ESIMS^2^ (452.2 🡪) *m/z* 434.2 (7), 311.3 (100); ‑ESIMS *m/z* 450.2 [M-H]^-^; -ESIMS^2^ (450.2 🡪) *m/z* 253.2 (100), 214.1 (1); +HRESIMS (High-resolution electrospray ionization mass spectrometry) *m/z* 452.2776 [M+H]^+^ (calcd for C_21_H_43_NO_7_P^+^, 452.2772, Δ = 0.9 ppm); -HRESIMS *m/z* 450.2620 [M-H]^-^ (calcd for C_21_H_41_NO_7_P^-^, 450.2626, Δ = -1.3 ppm).

**1-Palmitoleyl-2-hydroxy-sn-glycero-3-phosphoethanolamine (2-LPE) (2):** +ESIMS *m/z* 452.2 [M+H]^+^; +ESIMS^2^ (452.2 🡪) *m/z* 434.2 (100), 311.3 (42), 280.2 (1), 237.3 (3), 155.1 (2**)**; -ESIMS *m/z* 450.2 [M-H]^-^; -ESIMS^2^ (450.2 🡪) *m/z* 253.2 (100), 214.1 (3), 196.0 (3), 153.0 (1); +HRESIMS *m/z* 452.2772 [M+H]^+^ (calcd for C_21_H_43_NO_7_P^+^, 452.2772, Δ = 0.0 ppm); -HRESIMS *m/z* 450.2628 [M-H]^-^ (calcd for C_21_H_41_NO_7_P^-^, 450.2626, Δ = 0.5 ppm).

**Palmitoleic acid (3):** +HRESIMS *m/z* 255.2319 [M+H]^+^ (calcd for C_16_H_31_O_2_^+^, 255.2319, Δ = 0.3 ppm); -HRESIMS *m/z* 253.2171 [M-H]^-^ (calcd for C_16_H_29_O_2_^-^, 253.2173, Δ = -0.6 ppm).

**Palmitic acid (4):** +HRESIMS *m/z* 257.2475 [M+H]^+^ (calcd for C_16_H_33_O_2_^+^, 257.2475, Δ = -0.2 ppm); -HRESIMS *m/z* 255.2325 [M-H]^-^ (calcd for C_16_H_31_O_2_^-^, 255.2330, Δ = -1.6 ppm).

**Oleic acid (5):** +HRESIMS *m/z* 283.2630 [M+H]^+^ (calcd for C_18_H_35_O_2_^+^, 283.2632, Δ = -0.4 ppm); -HRESIMS *m/z* 281.2482 [M-H]^-^ (calcd for C_18_H_33_O_2_^-^, 281.2486, Δ = -1.4 ppm).
