# Supplementary material for: Trophosome of the Deep-Sea Tubeworm Riftia pachyptila Inhibits Bacterial Growth
Source: PLoS One. 2016 Jan 5;11(1):e0146446. doi: 10.1371/journal.pone.0146446 (PMC4701499; doi:10.1371/journal.pone.0146446)
Supplement: S1 Table — Probes were labeled on the 5’ end either with Cy3, FITC or Atto 488 to highlight symbionts in the trophosome and to analyze other bacteria on the tissue-sections. A NON-EUB338 probe was used as negative control. All hybridizations were carried out in 35% (vol/vol) formamide and counter-stained with DAPI. The Endoriftia oligonucleotide probe RifTO445 (Nussbaumer et al., 2006) is specific for the 16S rRNA of the Riftia pachyptila, Tevnia jerichonana and Oasisia alvinae symbionts as all three vestimentiferans share an identical (Tevnia) or nearly identical (Oasisia) 16S rRNA symbiont phylotype (Rif/Tev/Oas) (Feldman et al., 1997; Vrijenhoek et al., 2010; Gardebrecht et al., 2012). (DOCX) [file pone.0146446.s002.docx]

| **probe/primer** | **sequence** | **specificity** | **reference** |
| --- | --- | --- | --- |
| EUB I | 5’-GCT GCC TCC CGT AGG AGT -3’ | most bacteria (EUB I+II+III) | Amann et al., 1990 |
| EUB II | 5’-GCA GCC ACC CGT AGG TGT -3’ | most bacteria (EUB I+II+III) | Daims et al., 1999 |
| EUB III | 5’-GCT GCC ACC CGT AGG TGT -3’ | most bacteria (EUB I+II+III) | Daims et al., 1999 |
| RifTO445 | 5’-TCC TCA GGC TTT TCT TCC-3’ | *Rif/Tev/Oas* symbiont | Nussbaumer et al., 2006 |
| NON-EUB | 5’-ACT CCT ACG GGA GGC AGC-3’ | negative control | Manz et al., 1992 |

**Supplementary References**

1. Amann RI, Krumholz L, Stahl DA. Fluorescent-oligonucleotide probing of whole cells for determinative, phylogenetic, and environmental studies in microbiology. J Bacteriol. 1990; 172(2):762-70

2. Daims H, Brühl A, Amann R, Schleifer KH, Wagner M. The domain-specific probe EUB338 is insufficient for the detection of all Bacteria: Development and evaluation of a more comprehensive probe set. System Appl Microbiol. 1999; 22(3):434-444

3. Nussbaumer AD, Fisher CR, Bright M. Horizontal endosymbiont transmission in hydrothermal vent tubeworms. Nature 2006; 441(7091):345-348

4. Manz W, Amann R, Ludwig W, Wagner M, Schleifer KH. Phylogenetic oligonucleotide probes for the major subclasses of Proteobacteria: Problems and solutions. System Appl Microbiol. 1992; 15(4):593-600
