# Supplementary material for: Trophosome of the Deep-Sea Tubeworm Riftia pachyptila Inhibits Bacterial Growth
Source: PLoS One. 2016 Jan 5;11(1):e0146446. doi: 10.1371/journal.pone.0146446 (PMC4701499; doi:10.1371/journal.pone.0146446)
Supplement: S2 Table — TM: trophosome middle part, SM: skin middle part, SU: skin upper part, cryo: fixed in liquid nitrogen, ethanol: fixed in 100% ethanol. (DOCX) [file pone.0146446.s003.docx]

| sample | tissue | sample ID | year | specification, incubation | East Pacific Rise vent site | used for | fixation |
| --- | --- | --- | --- | --- | --- | --- | --- |
| 1 | trophosome | 1540 | 2011 | adult TM, freshly fixed | TICA^*^ | HPLC-MS, Inhibition assay | ethanol, cryo |
| 2 | trophosome | 1541 | 2011 | adult TM, freshly fixed | TICA | HPLC-MS, Inhibition assay | ethanol, cryo |
| 3 | trophosome | 1556 | 2011 | adult TM, freshly fixed | P-Vent N^*^ | HPLC-MS, Inhibition assay | ethanol, cryo |
| 4 | trophosome | 1542 | 2011 | adult TM, 12h cold | TICA | HPLC-MS, Inhibition assay | ethanol, cryo |
| 5 | trophosome | 1542 | 2011 | adult TM, 24h cold | TICA | HPLC-MS, Inhibition assay | ethanol, cryo |
| 6 | trophosome | 1542 | 2011 | adult TM, 144h cold | TICA | HPLC-MS, Inhibition assay | ethanol, cryo |
| 7 | trophosome | 1542 | 2011 | adult TM, 12h warm | TICA | HPLC-MS, Inhibition assay | ethanol, cryo |
| 8 | trophosome | 1542 | 2011 | adult TM, 24h warm | TICA | HPLC-MS, Inhibition assay | ethanol, cryo |
| 9 | trophosome | 1542 | 2011 | adult TM, 144h warm | TICA | HPLC-MS, Inhibition assay | ethanol, cryo |
| 10 | trophosome | 1533 | 2010 | juvenile, freshly fixed | Genesis^*^ | Inhibition assay | cryo |
| 11 | trophosome | 1532 | 2010 | adult TM, freshly fixed | Janine^*^ | Inhibition assay | cryo |
| 12 | trophosome | 1530 | 2010 | adult, freshly fixed | Genesis | Inhibition assay | cryo |
| 13 | trophosome | 1540 | 2011 | adult TM, 12h ethanol control | TICA | Inhibition assay | cryo |
| 14 | trophosome | 1540 | 2011 | adult TM, 24h ethanol control | TICA | Inhibition assay | cryo |
| 15 | skin | 1540 | 2011 | adult SU, freshly fixed | TICA | HPLC-MS, Inhibition assay | ethanol, cryo |
| 16 | skin | 1541 | 2011 | adult SM, freshly fixed | TICA | HPLC-MS, Inhibition assay | ethanol, cryo |
| 17 | skin | 1556 | 2011 | adult SM, freshly fixed | P-Vent N | HPLC-MS | ethanol |
| 18 | skin | 1533 | 2010 | juvenile, freshly fixed | Genesis | Inhibition assay | cryo |
| 19 | skin | 1532 | 2010 | adult SM, freshly fixed | Janine | Inhibition assay | cryo |

^*^Location of hydrothermal vent sites: TICA 9°50,404 N, 104°17,495 W; P-Vent N 9°50,2816 N, 104°17,732 W; Genesis 12°48,67 N, 103°56,45 W; Janine 12°48,585 N, 103°56,413 W
