# Supplementary material for: Trophosome of the Deep-Sea Tubeworm Riftia pachyptila Inhibits Bacterial Growth
Source: PLoS One. 2016 Jan 5;11(1):e0146446. doi: 10.1371/journal.pone.0146446 (PMC4701499; doi:10.1371/journal.pone.0146446)
Supplement: S3 Table — Absolute peak areas of the lipids 2-palmitoleyl-1-lyso-PE (1-LPE), 1-palmitoleyl-2-lyso-PE (2-LPE), palmitoleic acid (C16:1), palmitic acid (C16:0), and oleic acid (C18:1) obtained by HPLC-CAD analysis of the corresponding ethanol supernatants. N/A: data not available. (DOCX) [file pone.0146446.s004.docx]

|  | | **Inhibition zone [mm]** | | | | | **Peak area [pA*min]** | | | | |
| --- | --- | --- | --- | --- | --- | --- | --- | --- | --- | --- | --- |
|  |  | ***B. subtilis*** | ***L. welshimeri*** | ***F. johnsoniae*** | ***M. smegmatis*** | ***S. aureus*** | **1-LPE** | **2-LPE** | **C_16_:1** | **C_16_:0** | **C_18_:1** |
|  | retention time [min] |  |  |  |  |  | **28.0** | **29.0** | **41.4** | **45.3** | **45.7** |
| trophosome | 1540^a^ freshly fixed | 0.11 | 0.56 | 1.11 | 3.06 | 0.56 | 0.098 | 0.130 | 0.007 | 0.053 | 0.010 |
|  | 1541 freshly fixed | 0.28 | 0.28 | 1.67 | 0.28 | 0.11 | 0.089 | 0.148 | 0.067 | 0.128 | 0.167 |
|  | 1556 freshly fixed | 0.28 | 0.28 | 0.56 | 1.67 | 0.28 | 0.032 | 0.087 | 0.019 | 0.039 | 0.028 |
|  | *Average freshly fixed* | *0.22* | *0.37* | *1.11* | *1.67* | *0.32* | *0.073* | *0.122* | *0.031* | *0.073* | 0.068 |
|  | 1542 24h cold | 0.28 | 0.39 | 0.00 | 0.00 | N/A | 2.264 | 1.131 | 0.286 | 0.244 | 0.343 |
|  | 1542 144h cold | 0.80 | 1.11 | 0.00 | 0.56 | N/A | 1.248 | 0.852 | 0.240 | 0.262 | 0.338 |
|  | 1542 24h warm | 0.11 | 0.83 | 0.00 | 1.39 | N/A | 0.230 | 0.264 | 0.071 | 0.164 | 0.104 |
|  | 1542 144h warm | 1.67 | 1.94 | 2.22 | 0.00 | N/A | 0.120 | 0.389 | 5.135 | 0.551 | 4.129 |
| skin | 1540 freshly fixed | 0.00 | 0.00 | 0.00 | 0.00 | 0.00 | 0.001 | 0.008 | 0.007 | 0.041 | 0.011 |
|  | 1541 freshly fixed | 0.00 | 0.00 | 0.00 | 0.00 | 0.00 | 0.000 | 0.000 | 0.000 | 0.063 | 0.000 |
|  | 1556 freshly fixed | 0.00 | 0.00 | 0.00 | 0.00 | 0.00 | 0.000 | 0.000 | 0.000 | 0.042 | 0.000 |
|  | *Average freshly fixed* | *0.00* | *0.00* | *0.00* | *0.00* | *0.00* | *0.000* | *0.003* | *0.002* | *0.049* | 0.004 |

^a^ sample ID (see S2 Table)
